# Salivary Cortisol in the Diagnosis of Cushing Syndrome, Always More Than One!

**DOI:** 10.1210/jendso/bvaa109

**Published:** 2020-09-09

**Authors:** Maria Fleseriu

**Affiliations:** Pituitary Center, and Departments of Medicine (Endocrinology) and Neurological Surgery, Oregon Health & Science University, Portland, Oregon, USA

Cushing syndrome (CS) is a very complex disease with many comorbidities and an increased mortality if not appropriately treated. Even today, many patients experience a significant delay in diagnosis. Given that many clinical features overlap with other conditions, biochemical evaluation is essential. Salivary cortisol measurements have been performed since the 1960s, but use of late-night salivary cortisol (LNSC) as a reliable screening for endogenous CS was established 3 decades later [1]. Raff et al in 1998 reported that an elevated 2300-hour salivary cortisol identified 36 of 39 proven CS patients with 92% sensitivity [1]. If elevated urinary free cortisol (UFC) was also considered, sensitivity increased to 100% [1]. Rarely in life do results reach 100% particularly so in medicine; since initial studies, methods, and assays have changed over time, resulting in different reference ranges and as such yield differences in sensitivity and specificity. Notably, the upper limit of normal (ULN) reference range for the assay by Raff and colleagues [1] as measured by radioimmunoassay (RIA) was 3.6 nmol/L, with a low (3%) intra-assay coefficient of variation. ULN reference ranges are overall much lower as measured by high-performance liquid chromatography or liquid chromatography with tandem mass spectrometry (LC-MS/MS) than in antibody-based assays, which is essential for accurate clinical interpretation. Analytic assay issues have been reported both with direct immunoassay and LC (less so with LC-MS/MS). LNSC measured by LC-MS/MS using smaller saliva volumes (50 μl) and samples at usual bedtime instead of late-night samples have shown a good correlation in healthy adults with an LNSC (US Food and Drug Administration–approved) enzyme-immunoassay (EIA) [2].

Debono et al showed in 2016 that salivary cortisone had a better correlation with serum cortisol than salivary cortisol, especially for low serum cortisol levels [3]. Interestingly, although measured salivary cortisol and cortisone levels are free (nonbound) levels, one study in healthy volunteers showed a nonsignificant trend of higher salivary cortisol and cortisone levels in women with higher estrogen levels (pregnant women or those taking oral contraceptives) [4].

The Endocrine Society Clinical Practice Guideline [5] includes salivary cortisol, together with an overnight dexamethasone test (ODT) and UFC as initial testing for all types of CS. The guideline raises awareness that the effect(s) of sex, age, and coexisting medical conditions (diabetes, hypertension, and obesity) on LNSC values have not been fully characterized and that older males with comorbidities could have higher LNSC values even in the absence of CS [5].

The use of salivary cortisol and cortisone, especially as measured by LC-MS/MS, has exponentially increased overtime, not solely for late-night measurements, but also after ODT [4]. One recent study showed 95% (range, 75%-100%) sensitivity and 96% (range, 92%-99%) specificity for LNSC and 100% (range, 83%-100%) sensitivity and 94% (range, 89%-97%) specificity for LNSC cortisone [4]. Interestingly, although most studies show that LNSC measurements at 23:00 hours are essential, measurements an hour earlier at 22:00 hours did not seem to make a difference [4], a finding that could improve usage.

With more use comes more scrutiny. Most of the aforementioned studies were undertaken in patients at specialized pituitary centers, whereby sampling is enriched with CS patients, rather than with nonneoplastic hypercortisolemia patients. One remaining question is how specific LNSC could be in the general population (suspected of CS) when used for screening.

The study by Kannankeril and colleagues [6] prospectively evaluated the diagnostic performance of an enzyme immunoassay (EIA-F), salivary cortisol (LCMS-F), and cortisone (LCMS-E) in 1453 consecutive LNS samples from 705 patients with suspected CS. The study conclusions are very interesting and not totally surprising. The authors show that a majority of patients with 1 or more elevated LNSC or cortisone results did not have CS, and a single elevated level had both poor specificity and positive predictive value. Likewise, another important study finding was that LNSC (as measured by EIA), though a sensitive test for adrenocorticotropin (ACTH)-dependent CS, did not have the same value for ACTH-independent CS (adrenal adenoma). Furthermore, the authors suggest that neither LCMS-F nor LCMS-E improves the sensitivity of late-night EIA-F for CS.

From my perspective, the study highlights 2 important points. First, similar to other screening tests, biochemical screening for neoplastic hypercortisolism should take into consideration the pretest probability of CS. For example, one high value could be a false-positive test. This is extremely important because one does not want to overlook a CS diagnosis. However, an accurate diagnosis is essential to avoid unnecessary treatment. Second, and perhaps less well recognized, LNSC measurement is not a valuable screening tool in patients with an incidental adrenal nodule and possible mild autonomous cortisol excess (MACE). For these patients the ODT remains the test of choice.

Disease recurrence in Cushing disease (CD) patients (in remission post–initial surgery), is higher than initially thought and can reach 25% to 30% over a lifetime [7]. LNSC has been established as the first choice in assessing CD recurrence, and although variability can be an important pitfall, it is overall a better choice than 24-hour UFC, and can sometimes reveal abnormal results almost a year ahead of other tests [7].

More recently, because LNSC is a simple, convenient biomarker, it has been studied to assess treatment response in patients with CD. Observations from large studies [8] that used UFC normalization as an end point showed that LNSC displays a high (~50%) degree of intrapatient daily variability, not unlike UFC. However, patients treated with pasireotide LAR who achieved both normal LNSC and UFC levels showed the greatest clinical improvements, thus highlighting that one should measure both LNSC and UFC for a more comprehensive appraisal of response to medical treatment in CD patients [8].

In conclusion, over the last few decades, the role of salivary cortisol and cortisone measurements both in screening and monitoring of remission or medical treatment effectiveness has significantly increased. The major advantages of LNSC are noninvasiveness, relative reliability, independence from variations in plasma cortisol-binding globulin and dexamethasone metabolism, and easier use at home for patients in an outpatient setting. Furthermore, LNSC is preferred in the evaluation of suspected intermittent hypercortisolism when patients require many samples per week or month. Patients with abnormal sleep-wake cycles or diseases known to cause physiologic hypothalamic–pituitary–adrenal axis activation [5, 6] should not undergo LNSC testing, and the possibility of sample contamination (topical corticosteroids, blood) should be taken into account in cases with unexplained very high values.

However, as described in this excellent manuscript [6], the value of LNSC can diminish in screening of large populations with suspected CS, and results should be interpreted based on pretest probability. Screening for MACE in cases of adrenal adenomas should continue to rely on ODT, whereas plasma cortisol of less than 1.8 mcg/dL and greater than 5 mcg/dL are diagnostic for most patients, patients with cut-offs between 1.8 and 5 mcg/dL need to have additional confirmatory testing, and LNSC is not always reliable.

There is still work to do, especially in establishing clinically relevant reference intervals for all laboratories and increasing clinician awareness about the importance of pretest probability, any preanalytical error(s), and regarding testing/methods/assay differences that can significantly influence result accuracy. Finally, one always needs more than one patient sample and result(s) to determine and ascribe a CS diagnosis.

## Additional Information

***Disclosure Summary:*** M.F. has received research support as a principal investigator for Oregon Health & Science University from Novartis and Strongbridge and as an occasional scientific consultant to Novartis and Strongbridge.
